# Myocardial Accumulations of Reg3A, Reg3γ and Oncostatin M Are Associated with the Formation of Granulomata in Patients with Cardiac Sarcoidosis

**DOI:** 10.3390/ijms22084148

**Published:** 2021-04-16

**Authors:** Praveen Gajawada, Ayse Cetinkaya, Susanne von Gerlach, Natalia Kubin, Heiko Burger, Michael Näbauer, Carola Grinninger, Andreas Rolf, Markus Schönburg, Yeong-Hoon Choi, Thomas Kubin, Manfred Richter

**Affiliations:** 1 Department of Cardiac Surgery, Kerckhoff Heart Center, Benekestr. 2-8, 61231 Bad Nauheim, Germany; p.gajawada@kerckhoff-klinik.de (P.G.); a.cetinkaya@kerckhoff-klinik.de (A.C.); nataliajudo@aol.de (N.K.); h.burger@kerckhoff-klinik.de (H.B.); m.schoenburg@kerckhoff-klinik.de (M.S.); 2 Campus Kerckhoff, Justus-Liebig-University Giessen, 61231 Bad Nauheim, Germany; a.rolf@kerckhoff-klinik.de; 3 Universitätsklinikum Giessen und Marburg GmbH, Standort Marburg, Baldingerstr., 35033 Marburg, Germany; susanne.vongerlach@uni-marburg.de; 4 Medizinische Klinik und Poliklinik I, Klinikum der Universität München, Marchioninistr. 15, 81377 Munich, Germany; michael.nabauer@med.uni-muenchen.de (M.N.); Carola.Grinninger@med.uni-muenchen.de (C.G.); 5 Department of Cardiology, Kerckhoff Heart and Lung Center, Benekestr. 2-8, 61231 Bad Nauheim, Germany; 6 German Center for Cardiovascular Research (DZHK), Partner Site RhineMain, 60549 Frankfurt/Main, Germany

**Keywords:** chemokine, chemoattraction, macrophage, inflammation, interleukin-1 receptor antagonist, cell signaling, dedifferentiation, remodeling, myocarditis, heart failure

## Abstract

Cardiac sarcoidosis (CS) is a poorly understood disease and is characterized by the focal accumulation of immune cells, thus leading to the formation of granulomata (GL). To identify the developmental principles of fatal GL, fluorescence microscopy and Western blot analysis of CS and control patients is presented here. CS is visualized macroscopically by positron emission tomography (PET)/ computed tomography (CT). A battery of antibodies is used to determine structural, cell cycle and inflammatory markers. GL consist of CD68^+^, CD163^+^ and CD206^+^ macrophages surrounded by T-cells within fibrotic areas. Cell cycle markers such as phospho-histone H3, phospho-Aurora and Ki67 were moderately present; however, the phosphorylated ERM (ezrin, radixin and moesin) and Erk1/2 proteins, strong expression of the myosin motor protein and the macrophage transcription factor PU.1 indicate highly active GL. Mild apoptosis is consistent with PI3 kinase and Akt activation. Massive amounts of the IL-1R antagonist reflect a mild activation of stress and inflammatory pathways in GL. High levels of oncostatin M and the Reg3A and Reg3γ chemokines are in accordance with macrophage accumulation in areas of remodeling cardiomyocytes. We conclude that the formation of GL occurs mainly through chemoattraction and less by proliferation of macrophages. Furthermore, activation of the oncostatin/Reg3 axis might help at first to wall-off substances but might initiate the chronic development of heart failure.

## 1. Introduction

Systemic sarcoidosis is a rare granulomatous disease with a prevalence of 5–64 per 100,000 people and can vary considerably between ethnic groups, regions, sex and age [1,2]. It is regarded as an autoimmune disease triggered by the exposure of genetically susceptible individuals to antigens and affects various organs and tissues, especially lymph nodes, skin, eyes, and lungs. Clinical studies of patients with sarcoidosis have revealed that less than 5% show cardiac involvement [3]; however, several studies point out that the true incidence of cardiac sarcoidosis (CS) might be strikingly higher. A pathological study of 84 consecutively autopsied patients with systemic sarcoidosis showed 25% myocardial involvement [4]. A further analysis of 62 patients with known sarcoidosis but without reported cardiac documentation revealed a proportion of 40% with CS [5]. Despite its discovery almost 150 years ago and the enormous worldwide research efforts, the variable phenotypic presentations of sarcoidosis, its obscure etiology and its frequently unspecific treatment options still cause controversial debates in the scientific and clinical fields [6]. In addition, the healing effects of available treatments can vary between organs and tissues, indicating that various signaling cascades might be involved to different degrees in the development of sarcoidosis and its progression [7]. 

Similar to other organs, sarcoidosis of the heart is characterized by the formation of non-caseating granulomata. CS is difficult to distinguish from the less well formed necrotizing giant cell myocarditis and requires therefore a meticulous histopathological evaluation. The granuloma consists of an organized collection of mature mononuclear phagocytes and is surrounded by numerous infiltrating cells. Multinucleated giant cells may exist in the center of a granuloma, which are regarded to be formed by the fusion of macrophages. The clinical manifestations of CS include asymptomatic conduction abnormalities, but also ventricular arrhythmia and complete heart blockage [3]. While some patients recover from minimal cardiac symptoms, others experience fatal consequences such as massive loss of muscle structure and subsequent fibrosis. The major causes of lethal outcomes are progressive congestive heart failure and sudden death. Advances in imaging capabilities such as cardiac magnetic resonance imaging (MRI) and fluorodeoxyglucose (FDG)-PET are being increasingly used as diagnostic tools for both asymptomatic and symptomatic CS patients, but the tissue diagnosis of CS is challenging even when utilizing endomyocardial biopsies due to the sampling errors that occur due to patchy or focal infiltration. This might be the reason why despite the steadily increasing number of original research publications focusing on CS, published data obtained by high-resolution immunofluorescence analysis or Western blotting are almost not existing. In addition, due to the present unspecific immunosuppressive treatment of CS causing various side effects, innovative and targeted therapeutic approaches are in demand. Thus, the aim of this study is to identify new features of cardiac granulomata and determine potential pharmacological targets. 

## 2. Results

### 2.1. Patients and General Experimental Design

**Figure 1:**
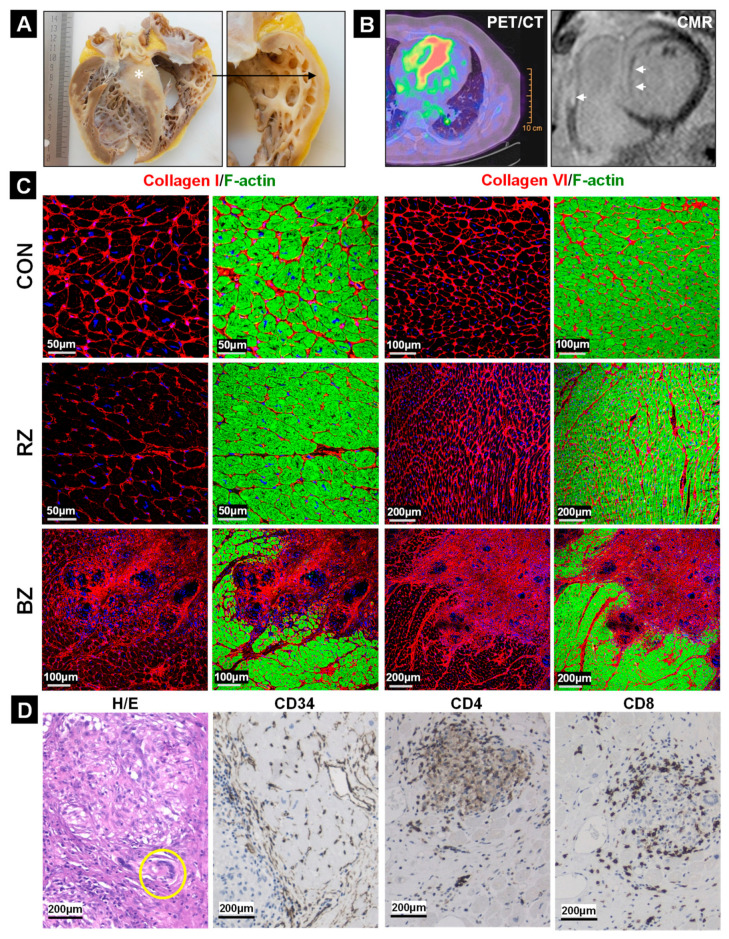
Loss of muscle mass, fibrosis and accumulation of immune cells are features of cardiac granuloma. (**A**) The macroscopic image of an explanted heart with cardiac sarcoidosis shows strong loss of muscle structure, wall thinning (magnified image indicated by a black long arrow) and massive fibrosis (white star). (**B**) ^18^F-FDG PET/CT image of a patient with cardiac sarcoidosis. Uptake of fluoroglucose indicates local inflammation in the heart and is shown in red. Cardiac magnetic resonance imaging (CMR) reveals marked septal and ventricular late enhancement (white arrows). (**C**) Staining of collagen I and VI mark fibrotic areas. For comparison, the control (CON) tissue is shown. Border zone (BZ) is the myocardial area surrounding the granuloma and remote zone (RZ) is the myocardium distant to granuloma. The nuclei are stained blue with DAPI. (**D**) Hematoxylin and eosin staining (H/E) display epithelioid granulomata with “horseshoe” like giant cells (yellow circle). The CD34 immunohistochemical image marks capillaries around the granuloma. Immunohistochemistry reveals CD4 and CD8 positive T cells mainly surrounding the granuloma. See also the Figure 2 fluorescence images.

The ejection fraction of patients with cardiac sarcoidosis (CS, Table 1) were measured to be 23.8 ± 4.1% and the hearts appeared dilated (Figure 1A) with no obvious coronary disease. Two patients showed pulmonary involvement and two suffered further from acute myocarditis. Since increased glucose metabolism is regarded as an indicator of inflammation, an increased uptake of the glucose analog ^18^F-FDG was visualized by a PET/CT [8] (Figure 1B). Cardiac magnetic resonance imaging (CMR) revealed septal and ventricular late enhancement (Figure 1B).

For the simplification of data interpretation, we have defined the remote zone (RZ) as the distal field of the granuloma with preserved cardiomyocytes, little fibrosis and few immune cells. We specify the border zone (BZ) as the area of the granuloma with neighboring cardiomyocytes. In order to obtain an overall image of giant cell granuloma, we utilized a battery of antibodies against structural, cell cycle, signaling cascades and chemokines which reflect cellular activities and might provide functional hints to pharmacological targets. Tissues samples from patients with aortic stenosis as well as preserved myocardial structure and ejection fractions (EF > 50%) served as controls (CON) [9]. The utilized antibodies, distributors, short explanations and abbreviations are listed in Table 2. An overall summary and interpretation of the results is given in Figure 7.

### 2.2. The Development of Granulomata (GL) Is Associated with Loss of Cardiomyocyte Mass and Scar Formation

Large areas of fibrotic tissue (white star) and muscle structure loss (black arrow) are clearly visible in a macroscopic image of a heart with CS (Figure 1A). The evaluation of myocardial tissue via the fluorescence determination of fibrillar collagen I and non-fibrillar collagen VI (Figure 1C) revealed regular arrangement of extracellular matrix products in the RZ but massive fibrosis in the BZ. In order to obtain a better picture of the damaged heart we performed immune staining, along with hematoxylin and eosin staining (Figure 1D). H/E visualizes the formation of large granulomata which are sometimes occupied by multinucleated giant cells (yellow circle). CD34 staining reveals vessels surrounding GL (Figure 1D) and CD4 as well as CD8 positive T-cells encircling granulomata (Figure 1D).

### 2.3. Macrophages in the Granuloma Show Mild Apoptosis and Are Highly Activated

**Figure 2:**
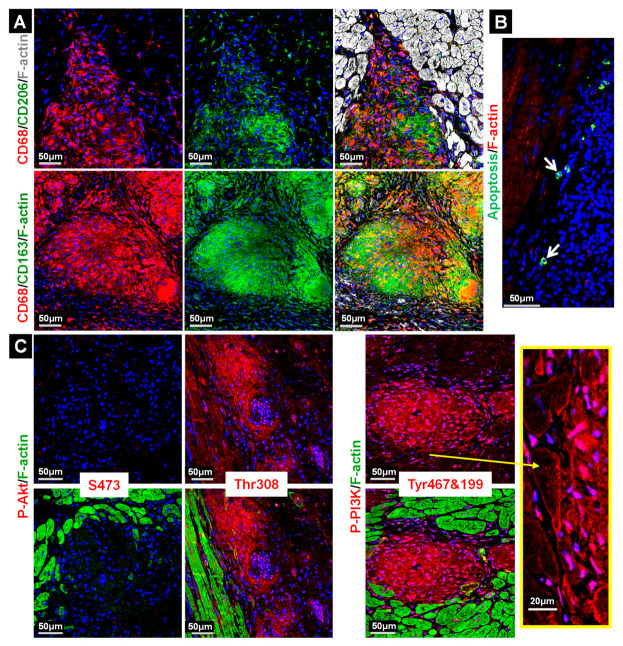
The PI3 kinase pathway is activated in macrophages of the granuloma and show mild apoptosis. (**A**) Confocal images of CD68^+^/CD163^+^/CD206^+^ stains demonstrate that the major cell pool in the granuloma consists of macrophages. (**B**) Mildly apoptotic cells were identified and are mainly located outside the granuloma. (**C**) The phosphoinositide 3-kinase (P-PI3K) and P-Akt/PKB (P-Akt) survival pathway is activated. Mainly threonine 308 (Thr308) but little serine 473 (S473) of the Akt kinase (P-Akt) is phosphorylated in granuloma. Phospho-sites are specified. Note that also surrounding cardiomyocytes show a PI3K activation (yellow arrow and enlarged yellow frame). The nuclei are stained blue with DAPI.

**Figure 3:**
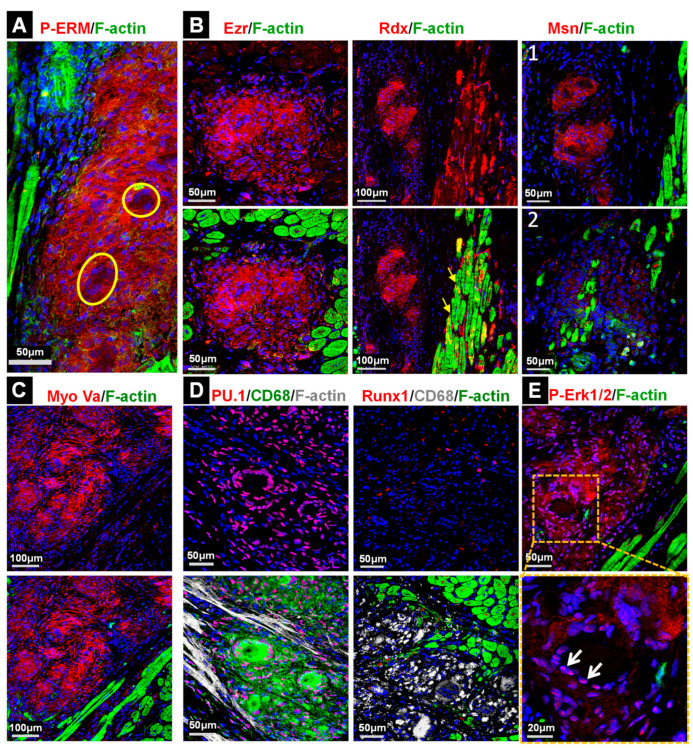
ERM (ezrin, radixin and moesin) proteins are strongly expressed and activated in granulomata (P-ERM). (**A**) P-ERM staining identifies highly activated GL. Giant cells are negative for P-ERM (yellow circles in enlarged images). (**B**) Ezrin (Ezr) reveals strong staining but radixin (Rdx) is also clearly present in GL. Moesin (Msn) shows various signal intensities indicating different qualities of granulomata (white 1 vs. white 2). Note the relocalization of radixin at the intercalated disc of cardiomyocytes (yellow arrows). (**C**) The molecular motor protein myosin Va is strongly expressed in granuloma. (**D**) Nuclear localization of the transcription factor PU.1 in macrophages is clearly visible but Runx1 appears to be absent. Note that the utilized antibody can recognize Run2 and 3. (**E**) Strong phosphorylation (P-Erk1/2) and nuclear localization (white arrows) of Erk1/2, as shown in the enlarged image, indicates activated macrophages. The nuclei are stained blue with DAPI.

Staining with CD68 revealed that the granulomata mainly consist of cells with a monocytic lineage (Figure 2A). Costaining with CD163 or CD206 showed that most phagocytes were macrophages (Figure 2A). The activation of the cell survival components PI3 kinase and Akt (Figure 2C,D), as well as the presence of few TUNEL-positive cells, is in accordance with mild apoptosis (Figure 2B). Threonine residue 308 (Thr308) in the Akt kinase was strongly phosphorylated, although little activation of serine 473 was observed. ERM proteins (ezrin, radixin and moesin) are a further indicator of cellular activation [10,11]. They stabilize the membrane and the actin cytoskeleton and are regarded as linker proteins. ERM proteins are strongly phosphorylated in macrophages of granulomata (P-ERM), whereas signals in giant cells are almost absent (Figure 3A). The analysis of individual ERM proteins (Figure 3B) revealed the major expression of ezrin (Ezr) and radixin (Rdx), while variations were found in the expression of moesin (Msn). Further indicators of activated granulomata include a high expression of myosin Va (Figure 3C), the strong nuclear localization of the transcription factor PU.1 (Figure 3D) and massive activation of the Erk1/2 signaling cascade (Figure 3E). Note that activated Erk1/2 translocates to the nucleus (Figure 3E). Myosin Va is an actin- and microtubule-based motor which regulates exocytosis and propels various cargos through cells, such as organelles, RNA and vesicles along filaments [12]. PU.1 is involved in differentiation and activation of macrophages [13]. The transcription factor Runx1, which can act in concert with PU.1 in macrophages, is hardly expressed in the GL (Figure 3D).

### 2.4. The Majority of Cell Cycle Marker Positive Cells in the Granuloma Are Non-Macrophages

**Figure 4:**
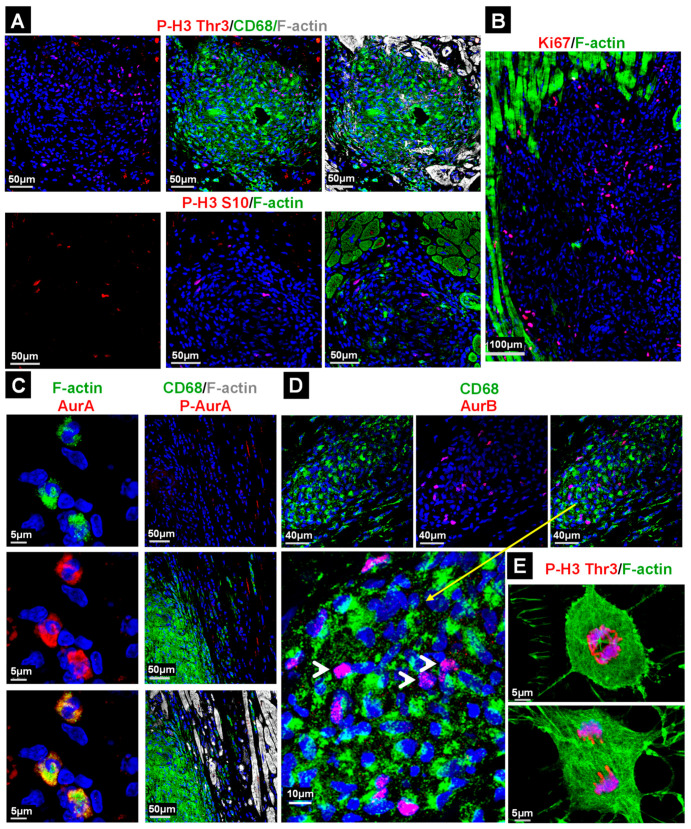
Cell cycle marker-positive macrophages reconstitute the minor cell pool in granulomata. (**A**) Phosphorylation of serine 10 (S10) and threonine 3 (Thr3) in histone H3 (P-H3) indicate cell cycle activation. (**B**) Ki67-positive cells are present in- and outside of granulomata. (**C**) Aurora A (AurA), filamentous actin (F-actin) and phospho-Aurora A (P-AurA, Thr288) highlight some cells in granulomata. (**D**) The costaining of Aurora B (AurB) with CD68 reveals mostly proliferating non-macrophages in GL (enlarged image, yellow arrow and white arrow heads). Note that none of the utilized cell cycle markers are indicative for a mitotic cell in of themselves. Since all used antibodies originate from rabbits, simultaneous staining of these markers was not performed. (**E**) Comparison images of mitotic non-cardiomyocytes in the culture. The nuclei are stained blue with DAPI.

The activation of Erk1/2, PI3K, Akt and ERM proteins suggests an involvement of proliferative activity in the formation of granulomata. Thus, we determined cell cycle-related proteins and their modifications (Figure 4). Phosphorylation of the histone-H3 residues Thr3 and S10 involved in chromosome condensation during mitosis occurred to different degrees in the granuloma (Figure 4A); however, since these sites might give signals independently of mitosis, the determination of further cell cycle markers such as Ki67, Aurora A and B and phospho-Aurora A/B/C (Thr288/Thr232/Thr198) is necessary. Aurora kinases are activated through phosphorylation and reach maximum activity and expression during the transition from G2 to the M phase [14].

Ki67-positive cells are clearly present in the granulomata (Figure 4B). The staining of Aurora A and filamentous actin demarcate potentially cycling cells in the granulomata (Figure 4C). In order to obtain further information regarding whether phagocytes are indeed positive for cell cycle markers, we performed costaining with CD68^+^ with phospho-histone-H3 (Thr3, Figure 4A), phospho-Aurora A/B/C (Figure 4C) and Aurora B (Figure 4D). Surprisingly, while all cell cycle markers were detected in the granulomata, only a few CD68^+^ cells were positive for P-H3 (Figure 4A), P-Aurora A/B/C (Figure 4C) and Aurora B (Figure 4D, white arrow heads) indicating that non-macrophages constitute a major pool of proliferating cells in GL. For comparison, cultured cycling non-cardiomyocytes are shown (Figure 4E).

### 2.5. Stress and Inflammatory Pathways Are Mildly Activated in the Granuloma and the Anti-Inflammatory Status Appears to Be Predominant

**Figure 5:**
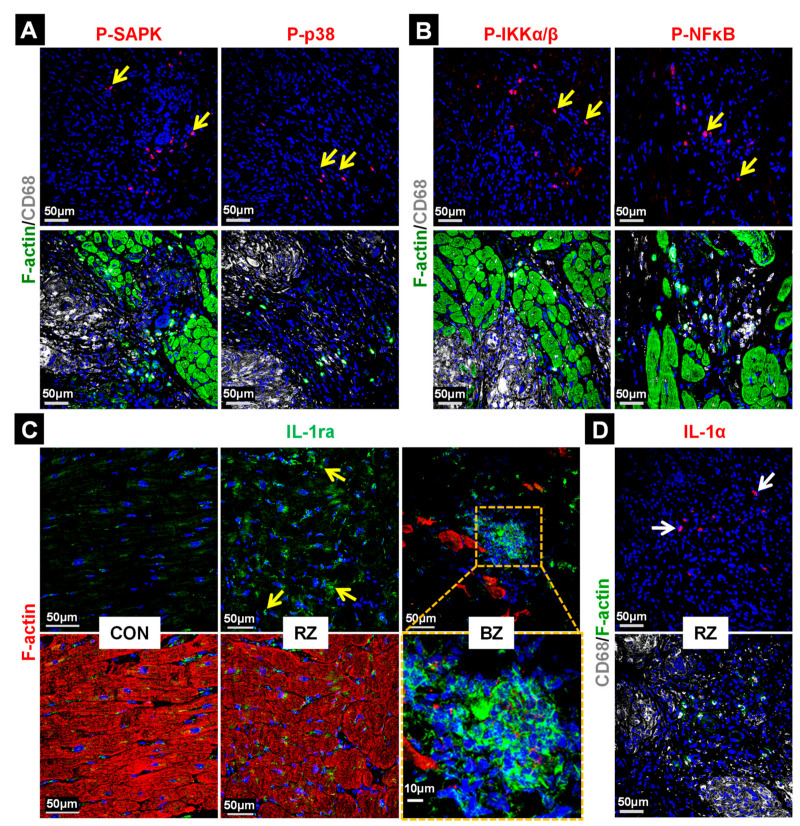
Stress and inflammatory cascades are mildly activated in granulomata. (**A**) Only a few infiltrating cells (yellow arrows) show the activation of the stress mediators SAPK (P-SAPK, Thr183/Tyr185) and p38 (P-p38, Thr180/Tyr182). (**B**) The inflammatory NFκB/IKKα/β pathway is also lowly activated (P-IKKα/β, Ser176/180; P-NFκB, Ser536). (**C**) In contrast, the anti-inflammatory interleukin-1 receptor antagonist (IL-1ra) is strongly expressed in macrophages of the granulomata (yellow arrow and enlarged image) and interstitial cells of the RZ and BZ (small yellow arrows). Comparable results were obtained with two different anti-IL-1ra antibodies (Table 2). (**D**) Interleukin-1α (IL-1α) is hardly present in the myocardium of patients with cardiac sarcoidosis (white arrows). The nuclei are stained blue with DAPI.

We considered whether stress and inflammatory pathways are activated in GL. Two central mediators of environmental challenges are mitogen-activated protein kinase p38 and stress-activated protein kinase (SAPK). These signaling cascades are activated by stress signals such as lipopolysaccharides and inflammatory cytokines rather than by mitogens and represent promising anti-inflammatory targets [15]. The activation of phosphorylation at Thr180 and Tyr182 in p38 (P-p38) and JNK at Thr183 and Tyr185 (P-JNK) was hardly detectable in GL (Figure 5A). Similarly, the phosphorylation of NFκB (Ser536; P-NFκBp65) and IKKα/β (Ser176/180; P-IKKα/β) is regarded to play a major role in inflammatory and immune response. The NFκB cascade is only activated in very few cells and mainly outside of GL (Figure 5B, yellow arrows). A further reason for the low activation of inflammatory pathways might be the strong presence of the interleukin-1 receptor antagonist in the RZ and BZ (IL-1ra, yellow arrows, Figure 5C) which blocks the receptor for IL-1α and β. Comparable data were obtained with two different IL-1 receptor antagonist antibodies (Table 2). In addition, interleukin-1α is hardly detectable. Taken together, these data demonstrate that the inflammatory responses were kept at low levels in patients with CS undergoing a transplantation.

### 2.6. Elevations of Oncostatin M Correlate with Dedifferentiated Cardiomyocytes and Increased Amounts of Reg3A and Reg3γ in the Border Zone

**Figure 6:**
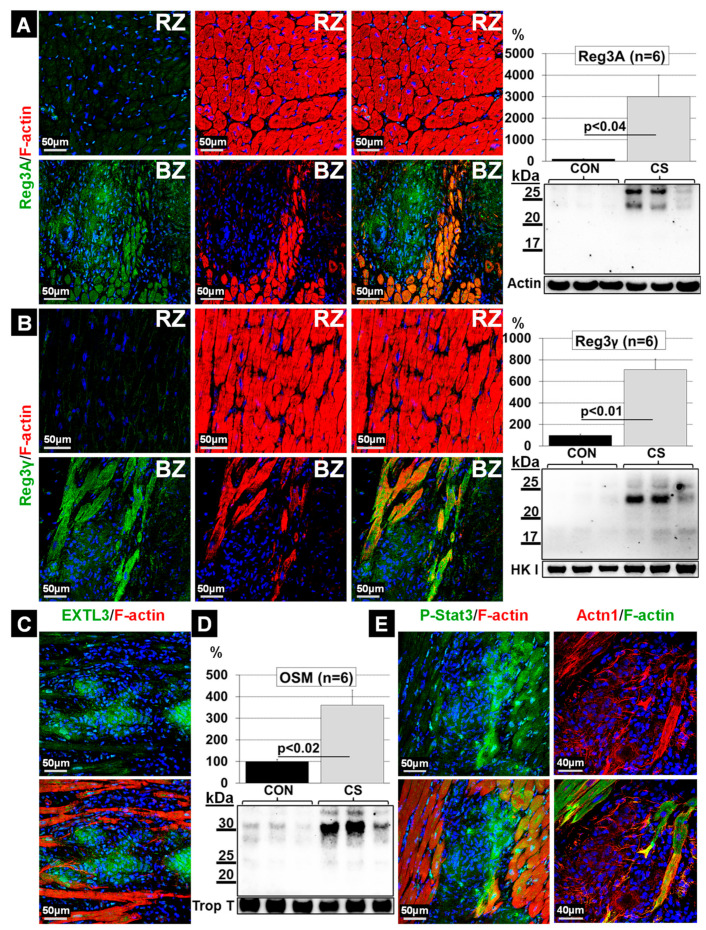
Increases in oncostatin M (OSM) correlate with elevations of the macrophage chemokines Reg3A and Reg3γ in the border zone. Myocardial tissue from six control patients (CON) and six patients with cardiac sarcoidosis (CS) were analyzed by Western blotting (WB) and immunofluorescence (IF). Troponin T (Trop T), actin and hexokinase I (HK I) serve as loading controls. (**A**) WB and IF images of Reg3A. (**B**) WB and IF images of Reg3γ. (**C**) IF demonstrates EXTL3 (a potential Reg receptor)-positive granulomata. (**D**) WB of OSM. (**E**) IF images of activated cardiomyocytes (P-Stat3, Tyr705). IF images show dedifferentiated non-muscle α-actinin 1 (Actn1) expressing cardiomyocytes in the BZ as well as the elongated and sharp shape of cardiomyocytes indicate furthermore OSM activity. The nuclei are stained blue with DAPI.

**Figure 7:**
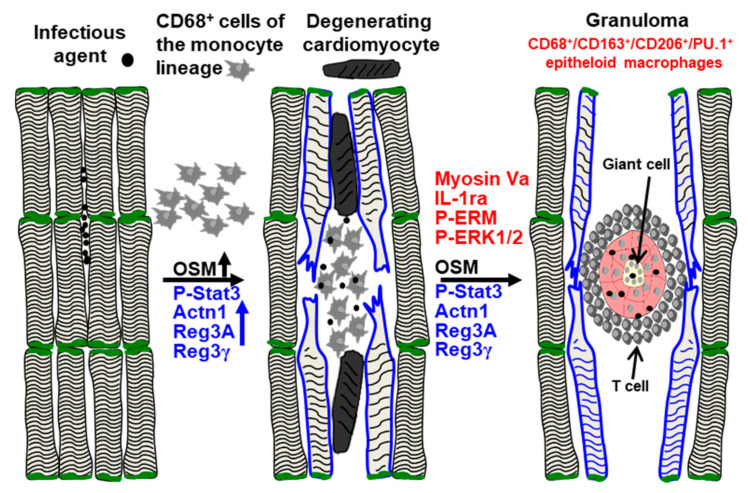
Hypothetical scheme of the formation of cardiac granulomata and the development of a vicious cycle by the activation of the OSM/OSMR/Stat3/Reg3 axes. Infectious signals evoke an infiltration of CD68^+^ monocytes/macrophages which release oncostatin M, which induces the activation and remodeling of cardiomyocytes (P-Stat3, Actn1, elongated sharp cell shape), and an accumulation of monocytes/macrophages through the release of regenerating islet-derived proteins (Reg3A and Reg3γ). The incomplete removal of infectious signals leads to chronic infiltration, the formation of highly active granulomata, cardiomyocyte degeneration and fibrosis in affected myocardial areas.

We considered whether chemoattraction might play a role in the formation of cardiac granulomata. Since regenerating islet-derived proteins (Reg3) have been shown to orchestrate macrophage infiltration in cell cultures, as well as in infarcted myocardia [16], we determined Reg3 expression levels in the BZ by immunofluorescence and Western blotting (Figure 6). Reg3A (Reg3β is the rat and mouse equivalent) and Reg3γ were massively expressed in the BZ (Figure 6A,B). Immunostaining revealed that the expression of Extl3 denotes a potential Reg receptor that could contribute to the chemoattraction of macrophages (Figure 6C) [17,18]. Since cardiomyocytes are the major source of Reg3 in a damaged myocardium, it is not surprising that the myocytes surrounding the granuloma are strongly positive for Reg3γ. Conversely, the macrophage-derived cytokine oncostatin M, which is a potent inducer of Reg chemokines [16], was markedly elevated in the myocardium (Figure 6D). Since the expression of regenerating islet-derived proteins in cardiomyocytes is dependent on Stat3 activation [16], the strong elevation of Reg3 proteins is in accordance with Stat3 phosphorylation (Figure 6E). Furthermore, the marked dedifferentiation of cardiomyocytes in the BZ, visible in the re-expression of the non-muscle α-actinin 1, and the typical elongated shape with a relocalization of radixin are also strong indicators of oncostatin M action (Figure 6E). An overall hypothetical scheme of the formation of granulomata is shown in Figure 7.

## 3. Discussion

The pathogenesis of sarcoidosis is still unclear. Non-auto and auto-antigens are regarded to trigger the infiltration of mononuclear phagocytes and T-cells, which exert initially inflammatory and immunoregulatory effects; however, progression of the disease is believed to be characterized by anti-inflammatory effects and by the proliferation of fibroblasts connected to tissue scarring [19]. Thus, early anti-inflammatory treatment of CS is essential to improve prognosis since its progression weakens the efficacy of an anti-inflammatory treatment strategy [20,21]. The absence of activated stress (P-p39, P-SAPK), as well as inflammatory pathways (P-NFκB, P-IKKα/β), indicates a non-inflammatory status of the myocardium obtained from patients with end-stage heart failure. Moreover, the high expression of the interleukin-1 receptor antagonist in GL is striking, as well as in the RZ. This antagonist is able to prevent activation of the NFκB pathway and stress kinases by blocking the competitive binding of interleukin-1 to its receptor [22]. Our observations might help to explain why an anti-inflammatory corticosteroid therapy is beneficial at an early stage in patients with preserved left ventricular function but less effective at an advanced condition of cardiac sarcoidosis. Thus, inflammatory and anti-inflammatory responses reflect subsequent phases in the development of cardiac sarcoidosis and consequently further thought requires distinct treatment strategies.

The formation of cardiac granulomata correlates with the strong loss of muscle mass, cardiomyocyte dedifferentiation/remodeling, ventricular thinning and massive fibrosis in patients with end-stage heart failure. Most phagocytes in GL show a tight epithelioid pattern and express PU.1, CD68, CD163 and CD206 and can therefore be classified as mature macrophages. There are several indications that granulomata are not quiescent entities but are still highly active and well-organized islands of macrophages in a failing heart. The strong presence of myosin Va, which regulates exocytosis and propels various cargos through the cell [12], the nuclear localization of the transcription factor PU.1, which is involved in differentiation and activation of macrophages [13], and the massive phosphorylation of ERM and Erk1/2 proteins are characteristic features. Phosphorylation of ERM proteins at the C-terminal region leads to activation and crosslinking of the plasma membrane to the actin cytoskeleton. Since ezrin, radixin and moesin play key roles in many biological processes such as growth, migration and morphogenesis [11], it appears likely that these proteins support monocytic infiltration and at a later stage the formation and maintenance of granulomata. In addition, the activation of the anti-apoptotic PI3K/Akt pathway might contribute to sustain the high activity of granulomata by suppressing cell death proteins. The ability of P-Akt to activate a number of proteins important for the polymerization of actin molecules might further contribute to migration and stabilization of the cytoskeleton in macrophages of the GL [23]. Interestingly, multinucleated giant cells which are regarded to arise from fusion of mature macrophages are negative for P-ERM. It is therefore intriguing to speculate that activated ERMs proteins stabilize the encapsulation of non-auto and auto-antigens in GL. 

The large number of accumulating macrophages in patients with CS raises questions about the origin of these immune cells. One possibility might be an in situ proliferation of resident macrophages. In order to address this question, we have investigated the cell cycle markers phospho-histone H3 (Thr3, S10), Ki67, Aurora A, Aurora B and the phosphorylated forms of Aurora A/B/C in granulomata. All of these markers were moderately present or activated in GL and no cell cycle-specific morphological appearance of P-H3 stains in macrophages was observed (for comparison see Figure 4E). In addition, a number of cell cycle marker-positive cells were negative for the monocyte-specific lineage marker CD68, indicating that significant amounts of these cells are non-macrophages. An alternative explanation to proliferation might be the chemoattraction of monocytes/macrophages. In order to clarify whether the formation of granulomata occurs via accumulation of chemoattracted macrophages we determined the regenerating islet-derived protein (Reg) family members Reg3A and Reg3γ in the myocardium of patients with cardiac sarcoidosis. Human Reg3A corresponds, based on homology, to the mouse and rat Reg3β, also named HIP protein, PAP and p23 [18,24]. For the first time, we show here the involvement of Reg3A and Reg3γ in the development of CS. Dedifferentiating cardiomyocytes surrounding GL express and release both Reg3A and Reg3γ; however, interestingly, a marked Reg3A stain was also seen in macrophages and this needs further study. Evidence for the involvement of Reg proteins in heart diseases has arisen from several studies. Increased expression of cardiac Reg1 was observed in patients who died because of myocardial infarction as well as in animal models of pressure overload and coronary artery ligation [25]. Increased Reg3γ expression was found in hearts of pressure overloaded rats [26] and elevated Reg3β levels were determined in animal models of experimental autoimmune myocarditis [27,28]. 

We have previously demonstrated that Reg family members (Reg1, Reg3β and Reg3γ) are released by cardiomyocytes via oncostatin M-induced Stat3 activation [16]. Mice with a deletion of the Reg3β chemokine or the receptor for OSM (OSMR) fail to orchestrate macrophage infiltration in the infarcted myocardium and show a strongly decreased viability [16]. The myocardial accumulation of monocyte/macrophage-derived OSM, along with the expression of Reg3A and Reg3γ in remodeling cardiomyocytes, emphasizes the prevalence of an OSM/OSMR/Stat3/Reg axis in the development of cardiac sarcoidosis. The activity of this interleukin-6 family member can be recognized by the relocalization of radixin, by the activation of Stat3, the elongated and partly sharp cardiomyocyte shape and the strong re-expression of Actn1. This non-muscle actinin is a reliable marker of cardiomyocyte remodeling and dedifferentiation [10,29,30,31]. Since the release of oncostatin M in the infiltrated myocardium is restricted to cells of a monocytic lineage and T-lymphocytes [30,32], invading phagocytes might fuel the OSM/OSMR/Stat3/Reg3 axis by increasing the number of OSM-secreting macrophages (see the hypothetical scheme in Figure 7). While this probably evolutionary-conserved mechanism to fight infection is initially protective, its chronic extension might lead to a vicious cycle. It has been shown in a mouse model with cardiac-restricted overexpression of the monocyte-chemotactic protein-1 (MCP-1) that chronic macrophage infiltration thereby increased OSM activity, leading to myocarditis and finally heart failure [33]. Knockdown of the OSMR in this transgenic animal strain strongly reduced cardiomyocyte remodeling, prolonged survival and attenuated the development of dilated cardiomyopathy [9,30,31,34]. Albeit, the MCP-1 induced myocarditis with its diffuse cardiac accumulation of infiltrates is different from CS, where macrophages form islands, the attenuation of OSM signaling might be a therapeutic approach to restrict the deleterious formation of granulomata.

Finally, we have to address several study limitations due to the scarcity of samples from patients with cardiac sarcoidosis, as well as the lack of myocardial biopsies at different stages of the disease. All analyzed patients were suffering from end-stage heart failure and one might think that the observed activated pathways play only a role at the terminal-stage rather than during earlier phases. While we presently cannot clear this point, there are several indications that the activation of these pathways is stage independent. First, in contrast to the degeneration of cardiomyocytes, macrophages appear to be highly viable, as can be seen by very few apoptotic cells and phosphorylation of PI3K/Akt indicating that granulomata are resistant to a “burn out” over time. Second, activation of ERM proteins might be essential for the formation of granulomata but also for their homeostasis since they play an important role in tissue and organ development [11]. Third, signaling pathways appear to be activated at various developmental stages of granulomata and can be recognized by different expression levels of moesin. We have also to consider for future research other myocarditis relevant chemokines or their receptors such as the monocyte chemotactic proteins [33] as well as the interleukin-7 receptor-α [35], which might play a role in the formation and maintenance of granulomata.

## 4. Materials and Methods

### 4.1. Study Population

Cardiac tissue from six patients with sarcoidosis (CS) and an ejection fraction (EF) of 23.8 ± 4.1% was obtained during transplantation (Table 1). Tissue obtained from six patients with aortic stenosis (AoSt) and preserved EF (>60%) requiring subvalvular myocardial resection within an aortic valve replacement served as control samples (CON). The clinical data of the CON are already published (Supplementary Table 1 from reference [9]. All experiments performed in this study comply with the Declaration of Helsinki and were approved by the respective responsible ethical committees. All obtained tissue samples were immediately flash frozen and kept at −80 °C until usage.

### 4.2. Fluorescence Microscopy

Fixation, staining and confocal microscopy were carried out as reported previously [35]. For hematoxylin and eosin staining, sections were fixed in paraformaldehyde and processed with standard methods. All utilized antibodies are listed and described in Table 2.

### 4.3. Statistical Analysis

For statistical analysis, GraphPad Prism 6 (GraphPad Software, San Diego, CA, USA) was used alongside Student’s *t*-test with Welch correction. *p*-values of <0.05 were taken as statistically significant.

## 5. Conclusions

The relatively low activation of the stress and inflammatory signaling cascades in cardiac granulomata corresponds to a strong expression of the anti-inflammatory interleukin-1 receptor antagonist. The formation of viable and very active granulomata faces dedifferentiating and degenerating cardiomyocytes in severely fibrotic areas. We hypothesize that high myocardial levels of oncostatin M and regenerating islet-derived chemokines might be associated with the chronic development of granulomata in the heart. The unregulated activation of the OSM/Reg3A/Reg3γ axis might lead to the development of a deleterious vicious cycle as summarized in Figure 7.

## Figures and Tables

**Table 1:** Clinical data of six patients with a cardiac granuloma who had undergone a heart transplantation (HTX). Five patients were female and one was male. All patients developed the phenotype of a dilated cardiomyopathy (DCM) and none showed signs of coronary heart disease (CHD). Two of them revealed a pulmonary involvement of sarcoidosis and further two suffered from acute myocarditis. Mean left ventricular ejection fraction (LVEF) was lower than 25%. Abbreviations are explained in the following. NYHA—New York Heart Association; PCI—percutaneous coronary intervention; CABG—coronary artery bypass grafting; HTX—heart transplantation; COPD—chronic obstructive pulmonary disease; LVAD—left ventricular assist device; AVS—aortic valve surgery; MVS—mitral valve surgery; biv ICD—biventricular implantable cardioverter-defibrillator; ACE—angiotensin-converting-enzyme; AT1—angiotensin II receptor; CRP—C-reactive protein; LDH—lactate dehydrogenase; SGOT—serum oxaloacetic transaminase; SGPT—serum glutamic pyruvic transaminase; CK—creatine kinase; NT pro BNP—N-terminal pro Brain Natriuretic Peptide; PTT—activated and partial thromboplastin time; INR—international normalized ratio; HIV—human immunodeficiency virus; LVESD—left ventricular end-systolic diameter; LVEDD—left ventricular end-diastolic diameter.

**Demographic and Risk Factors**	**Mean ± s.e.m.**	Sildenafil 20 mg (%)	17
Number of patients	6	ASS (%)	0
DCM (%)	100	Amiodaron (%)	67
CHD (%)	0	L Thyroxin (%)	17
Etiology of DCM	**2x pulmonary involvement**	Statins (%)	0
	**2x acute myocarditis**	**Laboratory parameters**	
Age (years)	48.2 ± 4.3	Sodium (mmol/L)	138 ± 1
Gender male (%)	17	Potassium (mmol/L)	4.1 ± 0.2
NYHA class	3.5 ± 0.5	Creatinine (mg/dL)	1.06 ± 0.2
Body height (cm)	169 ± 2.9	Hemoglobin (g/dL)	12.2 ± 1
Weight (Kg)	60.3 ± 4.2	Hematocrit (%)	36.2 ± 4.1
BMI (kg/m2)	21 ± 1	CRP (mg/L)	0.8 ± 0.4
Prior myocardial infarction (%)	0	Leukocytes Ts/µL	6.5 ± 0.5
Prior PCI or CABG (%)	0	Total cholesterol (mg/dL)	196 ± 26
Prior smoking (%)	0	LDH U/L	265 ± 41
Prior hypertension (%)	17	SGOT (IU/L)	37.2 ± 13
PRIOR COPD (%)	17	SGPT (IU/L)	36.4 ± 16.6
Prior diabetes mellitus (%)	0	CK U/L	47.7 ± 6.6
Thrombocytopenia (%)	0	NT-pro BNP pg/mL	5580 ± 4021
Prior hypercholesterolemia (%)	0	Quick (Thromboplastin time) (%)	74.2 ± 8.3
Pre CABG (%)	0	PTT (s)	29.5 ± 1.9
pre HTX LVAD (%)	17	INR	1.2 ± 0.15
pre HTX AK-OP (%)	17	Hepatitis B & C; HIV	negative
pre HTX MK-OP (%)	0	**Echocardiographic characteristics**	
pre HTX biv ICD (%)	67	LVEF (%)	23.8 ± 4.1
Cardiac arrhythmia (%)	50	LVESD (mm)	56.6 ± 6
**Comedication**		LVEDD (mm)	68 ± 4.8
Beta blocker (%)	50	**Hemodynamic parameters**	
ACE inhibitor and/or AT1 antagonist (%)	83	Cardiac index (L/min*m)	23.8 ± 4.1
Diuretic (%)	100	Pulmonary vascular resistance (dynes*s/cm5)	206 ± 53
Digitalis (%)	17	Pulmonary capillary wedge pressure (mmHg)	26.4 ± 2.2
Aldosterone antagonist (%)	100	O2 saturation (%)	63.6 ± 3

**Table 2:** List of antibodies and analyzed proteins. Catalog number (Cat ID), target of the antibody, phosphorylation site (phospho-sites), description/function, host, used abbreviations and distributing companies are listed.

Cat ID	Target	Phospho-Sites	Description/Function	Antibody	Company
C2456	Collagen I	-	Extracellular matrix product	Mouse	Sigma
600-401-108	Collagen VI	-	Extracellular matrix product	Rabbit	Rockland
F0766	CD4 (Clone MT310)	-	T lymphocyte marker	Mouse	Dako
MA5-12259	CD4 (Clone4B12)	-	T lymphocyte marker	Mouse	Thermo-Scientific
IS623	CD8 (Clone C8/144B)	-	T lymphocyte marker	Mouse	Dako
108M-94	CD8 (Clone C8/144B)	-	T lymphocyte marker	Mouse	Cell Marque
134M-14	CD34 (Clone QBEnd/10)	-	Endothel/hematogenic progenitor cells	Mouse	Cell Marque
M0718	CD68 (Clone EBM11)	-	Macrophage marker	Mouse	Dako
ABIN741570	CD163	-	Macrophage marker	Rabbit	Bioss
123003	CD206 (Clone MR5D3)	-	Macrophage marker	Rat	BIOZOL
3149	P-Ezrin/Radixin/Moesin	see below	Cytoskeletal/membrane linker	Rabbit	Cell Signaling
3145	Ezrin	Thr567	Cytoskeletal/membrane linker	Rabbit	Cell Signaling
2636	Radixin	Thr564	Cytoskeletal/membrane linker	Rabbit	Cell Signaling
3150	Moesin	Thr558	Cytoskeletal/membrane linker	Rabbit	Cell Signaling
ab76543	PU.1/Spi1	-	Transcription factor	Rabbit	Abcam
ab207254	Runx 1,2,3	-	Transcription factor	Rabbit	Abcam
3402	Myosin Va	-	Molecular motor protein	Rabbit	Cell Signaling
9145	P-Stat3	Tyr705	Signaling cascade	Rabbit	Cell Signaling
4370	P-p44/42 MAPK	Thr202/Tyr204	Signaling cascade	Rabbit	Cell Signaling
4511	p-p38 MAPK	Thr180/Tyr182	Stress signaling cascade	Rabbit	Cell Signaling
4668	P-SAPK/JNK MAPK	Thr183/Tyr185	Stress signaling cascade	Rabbit	Cell Signaling
2697	P-IKKα/β	Ser176/180	Inflammatory signaling cascade	Rabbit	Cell Signaling
3033	P-NF-κB p65	Ser536	Inflammatory signaling cascade	Rabbit	Cell Signaling
ab207254	P-PI3 Kinase (p85α & γ)	Tyr467&199	Survival signaling cascade	Rabbit	Abcam
4060	P-Akt	Ser473	Survival signaling cascade	Rabbit	Cell Signaling
13038	P-Akt	Thr308	Survival signaling cascade	Rabbit	Cell Signaling
MAB200	Interleukin-1α	-	Inflammatory cytokine	Mouse	R&D Systems
ab124962	Interleukin-1 receptor antagonist	-	Blocks Il-1 inflammatory signaling	Rabbit	Abcam
ab175392	Interleukin-1 receptor antagonist	-	Blocks Il-1 inflammatory signaling	Rabbit	Abcam
AF-295	human Oncostatin M (OSM)	-	Cytokine of the IL-6 family	Goat	R&D Systems
MAB5965	human Reg3A	-	Chemokine (macrophage)	Mouse	R&D Systems
AP5606c	human Reg3γ	-	Chemokine (macrophage)	Rabbit	Abcepta
9701	P-Histone 3	Ser10	Cell Cycle marker	Rabbit	Cell Signaling
9714	P-Histone 3	Thr3	Cell Cycle marker	Rabbit	Cell Signaling
14475	Aurora A	-	Cell Cycle marker	Rabbit	Cell Signaling
3079	P-Aurora A/B/C	Thr288/Thr232/Thr198	Cell Cycle marker	Rabbit	Cell Signaling
3094	Aurora B	-	Cell Cycle marker	Rabbit	Cell Signaling
ab16667	Ki67	-	Cell Cycle marker	Rabbit	Abcam
ab68194	α-actinin-1	-	non-muscle α-actinin-1	Rabbit	Abcam
A7811	α-actinin-2 (EA-53)	-	sarcomeric α-actinin-2	Mouse	Sigma
2024	Hexokinase 1	-	Serves as loading control	Rabbit	Cell Signaling
ab8295	Troponin T	-	Serves as loading control	Mouse	Abcam

## Data Availability

The data presented in this study are available in the article.
